# Controlling the Uncontrollable: Patient Safety and Medication Management From the Perspective of Registered Nurses in Municipal Home Health Care

**DOI:** 10.1177/23333936221108700

**Published:** 2022-07-07

**Authors:** Therése Bielsten, Elzana Odzakovic, Agneta Kullberg, Jan Marcusson, Ingrid Hellström

**Affiliations:** 1 Jönköping University, Jönköping, Sweden; 2 Linköping University, Linköping, Sweden; 3 Ersta Sköndal Bräcke University College, Stockholm, Sweden

**Keywords:** home health care, medication management, information transfer, patient safety, registered nurse, Sweden

## Abstract

Most adverse events in health care are related to medication management and they are almost always preventable. Increased knowledge of patient safety related to medication management in home health care is an urgent issue to provide safe care for all patients regardless of where the health care takes place. This study explored patient safety within medication management in municipal home health care. Vignettes were used as stimulus during qualitative interviews with registered nurses. Three main themes with related subthemes were identified as challenges to patient safety within medication management in home health care: (1) challenges to information transfer, (2) challenges related to delegation, and (3) challenges of advanced medical treatments in the home. The issue of transfer of information permeated our findings. Coordinating medications, delegating tasks, along with more advanced care require clear communication between care providers to be compatible with patient safety within medication management in home health care.

## Introduction

Home health care is becoming more extensive and advanced in Sweden and globally. The rise in home health care is related to the decrease in institutional care and the increasing number of older people worldwide (Genet et al., 2011; Tarricone & Tsouros, 2008), and it is also related to individual preferences for being cared for in the home environment. In Sweden, there is an ongoing structural change in the health care system which implies that health care is becoming even more decentralized from the hospitals with the aim to provide patients health care in their communities and where they live their lives. The structural change is called “Nära vård” in Swedish (“Close care” directly translated) and lacks a distinct definition but is described as the broad base with primary care as hub. Care that is closest at hand for the patient geographically, with the highest availability, and which offers a broad competence based on different professions. The purpose of “Nära vård” is for care to be organized and based on the patient’s needs and conditions to a greater extent (Swedish Association of Local Authorities and Regions [SALAR], 2022). This means that there is a need to highlight and examine patient safety aspects of care provided outside the hospital to gain knowledge of preventable adverse events and to ensure quality of care.

The organization responsible for providing home health care differs among countries in terms of what is provided and to whom (Genet et al., 2011). The main part of the responsibility for home health care in Sweden lies with the municipalities up to registered nurse level. County councils provide the medical resources. The municipalities (290 in Sweden) and regions (21 county councils) are co-responsible for establishing a care plan for each patient, which means that there must be effective collaboration and transfer of information to provide patients with high-quality care. Patient safety in general has been well highlighted in hospital care where the environment has the structural conditions to practice patient-safe care, however, more research is needed on patient safety in home health care (Madigan, 2007; Sears et al., 2013; Yu et al., 2016), where the environment is less foreseeable. Patient safety in Sweden is defined as “protection against adverse events,” and the Swedish definition of adverse events is “suffering, discomfort, bodily or mental harm, illness or death caused by the health care system, and which is not an inevitable consequence of the patient’s condition” (National Board of Health and Welfare, 2005).

Medication error adverse events are well known threats to patient safety globally (Harrison et al., 2013; Sears et al., 2013), and are the third leading cause of death in the US (Makary & Daniel, 2016). Six hundred medical charts were reviewed for adverse events in a Swedish study, and the results showed that 37.7% of people receiving home health care had been affected by at least one adverse event, and that 75% of these could have been prevented (Schildmeijer et al., 2018). The most frequently occurring adverse events in home health care, according to the study above and Lindblad et al. (2018) and National Board of Health and Welfare (2016) were medication management errors.

Patients in home health care often have multi-morbidity, which implies that they have complex care needs (Haltbakk et al., 2019). For this reason, home health care in Sweden involves close collaboration with social care, where the nurse directs health and medical care around the patient. Thus, registered nurses delegate tasks to assistant nurses and social care staff due to the high numbers of people receiving home health care, such as, and perhaps above all, the administration of medications. Delegating means that nurses give unlicensed care staff the right to do a task that is, by law, to be performed by a registered nurse. However, the nurse is personally responsible for the delegated task and that the receiver of the delegated task has the conditions, competence, and patient safety knowledge necessary to perform the task (National Board of Health and Welfare, 2017).

A review of the literature showed that authors in the field of patient safety highlight the issue of patient safety culture in home health care. A small number of recent studies available (2018–2020) discuss the need for high quality care in home health care owing to the increasing numbers of older people (worldwide) who receive more advanced health care in their homes (e.g., Kollerup et al., 2018; Lindblad et al., 2018; Raimondi, 2019). This study contributes to greater knowledge about medication management and patient safety, and it highlights potential threats to patient safety in municipal home health care.

## Aim

The study explored patient safety within medication management from the perspective of registered nurses in municipal home health care.

## Methods

Drawing on Schuetz (1953) phenomenologically based sociology we used his framework of first- and second-order constructs in an attempt to gain understanding of the meaning of real-life events described by nurses working in home health care. To construct a common understanding of a phenomenon, Schütz proposes an intersubjective process that goes from subjective meaning (first-order construct) to objective meaning (second-order constructs). The process aims to result in a reconstruction of the contextual social world to generate an understanding of the phenomenon on a common level-shared and understood in a common social world (Schuetz, 1953). Inspired by Schütz’s (1973) framework of first- and second-order constructs we aimed to gain first-order information of the experiences on patient safety and medication management through vignette-based interviews with nurses in home health care.

### Development of Vignettes and Interviews

Vignettes are used as tools in research and are descriptions of people or events in relation to a construct or idea (Spector et al., 2016). Hughes and Huby (2004) have described vignettes as a stimulus that refers to text and images that research participants are to respond to. As a first step to develop the vignettes, we reviewed the 274 pages of fieldnotes from the study where registered nurses were shadowed during their working days in municipal home health care (Odzakovic et al., 2021) and distinguished notes related to medication management. We chose to use vignettes from the fieldnotes of shadowing because they were drawn from real life events reflecting the local context and meaning, and therefore considered to arouse reflection and provide credible descriptions of similar situations and comparisons (Barter & Renold, 1999). A total of 15 vignettes were presented to the nurses and displayed one by one on a screen in a meeting room at their units. Examples of vignettes used in this study are displayed in Table 1. When the vignettes were displayed to the nurses, the first author asked open questions, such as “What do you think of when you see this vignette?” and “Is this a common situation in home health care?” Follow-up questions were then asked, depending on the nurses’ responses to the vignettes. The interviews varied from 60 to 80 minutes. Interviews were audio recorded and transcribed verbatim by the first author.

**Table 1.** Examples of Vignettes From Fieldnotes.

Example of vignettes
Whether or not assistant nurses should be allowed to dose medications has been discussed, but no decision has been made
The social care managers sometimes require us to approve (for delegation) the personnel they send . . . we cannot just approve the person, for example if sh/e is unable to read or write.
Home care cannot access all hospital / health care center notes or medication lists
I must put on the patch today (morphine), even though the patient does not need it because no one from the health care center has responded my messages despite several weeks in between.
xx says that it is not uncommon for them to receive the report after the patient has been sent home and that medication lists have not been faxed to us from the hospital.

### Context and Procedure

The present study took place during 8 weeks in three home health care units of three municipalities in the county of Östergötland, Sweden. Six registered nurses, of which two were specialist nurses, participated. They had been registered nurses from 6 to 15 years. Their ages varied between 36 and 56 years of age. The inclusion criteria for this study were registered nurse for a minimum of 1 year and employed by a home health care unit in the county of Östergötland, Sweden.

We initiated the recruitment process by sending out emails to the care managers of two home health care units of two municipalities. From the first unit, three nurses declared their interest in participating in the study, but only one nurse completed the participation. From the second unit three nurses declared interest and they all completed participation. Due to the recruitment issues, the first author contacted the care manager of one more home health care unit of another municipality in Östergötland. Four nurses from that unit declared interest in participating in the study of which two completed participation. The nurses from the three units announced that they did not have time to leave their workplace for interviews. For this reason, the interviews could not be held with a mixed group of nurses from different units.

## Analysis

Data from the interviews were sorted using the six steps of thematic analysis by Braun and Clarke (2006). The transcribed interviews were read several times by the first author and at least once by the members of the research group as a first step. The first author highlighted text sections in the second step and made notes in the margins of the raw data to gain an overview of the data. The data were coded and grouped in the third step to organize it and to form initial subthemes. Data were inductively coded from the bottom up to be as close as possible to the nurses’ experiences. The subthemes were developed and discussed in the research group in the fourth step. Subthemes were clustered together to form main themes representative of each cluster in the fifth step. Thus, the analysis was based on the nurses’ subjective meaning which through abstraction could be presented at a higher level of objective meaning through subthemes and main themes. An example of the analysis process is displayed in Table 2. The data were reported in this article in the sixth step.

**Table 2.** Example of the Analysis Process.

Raw data	Subtheme	Main theme
*The hospital might call the telephone queue system and say that this person will need wound dressing, and maybe already at that point, you would want a copy of the care plan for wound dressing and the medications that were newly inserted. After all, that transition can be vulnerable if it does not flow correctly.*	Trying to control the uncontrollable	Challenges to information transfer
*I perceive it as a problem in health care, in general. I think we should have the same documentation system everywhere, and I think that nurses and assistant nurses should be able to read the same things. . . . and I think that patient safety, if it is to be patient safe, surely it should be the same system all over Sweden.*		
*. . .some health care centers do not even want to meet the nurses at home health care, and then they make changes . . . and no one informs you about them with the risk that you might be dosing the medication wrong at home because you didn’t know . . .*		

All members of the research group were involved in the analysis to ensure consistency and data-anchored findings. The nurses were contacted and given the opportunity to read the interviews to confirm that we had understood their first-order information correctly. Three nurses gave feedback but did not want to revise anything in the interviews. During the analysis process, it was of utmost importance to preserve the subjective meaning of the nurses’ work-related life world. The credibility, of second-order constructs depends on whether the researchers have succeeded in preserving the subjective meaning of first-order information. Schütz (1973) three postulates *Logical Consistency*, *Subjective Interpretation*, and *Adequacy* guided us to gain trustworthiness of our second-order constructs. The vignettes anchored in real-life events, as described by nurses working in home health care was compatible with *logical consistency* linked to the nurses’ daily work. Recognition of situations through the vignettes enabled a *subjective interpretation*, also at a level of intersubjectivity through the nurses mutual understanding of the specific contextual social world. Further, the second-order construct must correspond to the nurse’s experiences and be comprehensible and recognizable to them in order to preserve, in accordance with *adequacy*, the subjective meaning (Schütz, 1973).

## Ethical Considerations

The study was conducted in accordance with the Declaration of Helsinki (World Medical Association, 2013). Ethical approval for this study was granted by the Regional Ethical Review Board in Linköping, Sweden, and was assigned the following code: 2018/496-31. Oral and written consent were collected from the nurses, and they were informed that they could withdraw from the study without reason. The data collected were encoded and processed confidentially. Personal data were processed in accordance with the Data Protection Regulation (EU) 2016/67. and the supplementary Swedish regulation SFS 2018: 218. We initially ensured that the vignettes did not reveal any information about participants or the home health care unit and the same routine for the protection of participants and home health care units was applied to this study to ensure that no vignettes or citations would be able to be traced to any individual or his or her personal circumstances, or to the home health care unit. We reflected on the possible risks for the participants in this study. We identified a risk that meant that the vignettes could be perceived as negatively charged. The participants were informed that the vignettes were taken out of reality and were not intended to judge right or wrong action. Participation in this study can be seen as an advantage for the nurses and the organization they worked in when they had the opportunity to discuss different scenarios and reflect on them, which in turn can be considered an opportunity for quality improvement in the specific organizations.

## Findings

Three main themes with related subthemes were identified as challenges to patient safety within medication management in home health care: (1) challenges to information transfer with the subthemes “*The hunt for the medication list*” and *“Unclear division of responsibility between health care providers”* (2) challenges related to delegation with the subthemes “*Conveyor-belt delegation*” and “*The risks of extended delegation of tasks*” and (3) challenges of advanced medical treatments in the home.

### Challenges to Information Transfer

A major problem that threatened patient safety was that municipal home health care and hospitals, along with health care centers, did not have the same medical record system. The fact that the nurses did not have full access to the medical record system caused problems and a sense of trying to control the uncontrollable. One of the main issues that created a sense of lack of control was the nurses limited access to medication prescription lists (hereinafter medication lists), when patients were admitted to the hospital, or had an appointment at the hospital or at the health care center. It was not uncommon that the nurses did not know when their patients had been on such a visit, what the visit was about, or if changes were made to the patient’s medication. This led to difficulties in maintaining patient safety.

Sometimes the nurses did not even know if their patients had been admitted to the hospital or if they had been discharged. To increase patient safety, the nurses tried to secure this information by ensuring that it was noted in the hospital’s medical record that the patient had been registered with home health care. Despite their efforts, it was relatively common to get the report from the hospital after the patient had already been discharged or when social care staff called and told them that the patient was at home *“Yes, you have no idea, the social care staff call and say, “you know he is at home, don’t you?” Uh, no, nobody told me that”* (*nurse 1*).

Occasionally, relatives who were with the patient at the hospital received the report prior to discharge instead of the nurse at home health care. One nurse said that staff at the hospital could be surprised when s/he questioned reports to relatives and when s/he demanded that epicrisis and medication lists be faxed to them.

*. . . it does not feel reasonable that I should dose based on what a sister has written in the middle of the night, but they* (staff at the hospital) *did not think that was weird at all. But you do not work like that in a hospital, you would not . . . if a sister to a patient comes and says, “take this drug, the doctor has said you are to give it to my brother,” you don’t do that, but that’s what happens to me with a handwritten note.* (nurse 3)

Some of the nurses felt that the transfer of information worked relatively well at the health care centers, but they requested more foresight on the part of the hospitals because it takes time to obtain medications and materials and to coordinate home visits for home health care. The sometimes-insufficient transfer of information contributed to hospital discharge being a particularly vulnerable situation.

The patients from two of the home health care units were listed at several different health care centers. The patients of one nurse had eight different health care centers. These health care centers also had different routines for their feedback on patients, if they gave any feedback at all to the nurses in home health care.

*Some health care centers do not even want to meet the nurses at home health care, and then they make changes . . . and no one informs you about them with the risk that you might be dosing the medication wrong at home because you didn’t know . . .* (nurse 4)

The nurses also stated that there were shortcomings in the transfer of information within their own medical record system, which they shared with assistant nurses and social care staff. If they wanted the assistant nurses and social care staff to be able to read their notes, they had to draw up a care plan for a problem (which could involve medication management) which they then distributed by sending it to the relevant staff; they could not just write a note under the relevant keyword in the medical record to make the note visible to the care staff.

#### The hunt for the medication list

The limited access to medication lists became a start of a “hunt.” The hunt could be lengthy and was described as time ineffective. The nurses were dependent on a nurse from a different health care provider to access the medication lists, often a nurse at the hospital or the health care center. *“. . . to get contact with all the instances . . . the medication lists are a nagging problem, and they can say “now they are calling from home care again about this list”* (nurse 3).

As an effort to increase patient safety, the nurses worked to get all patients on “Apodos” (to receive pre-packaged doses from the pharmacy) because then they had access to an accurate medication list via the Apodos system. When it came to patients who did not use Apodos (where the nurse dosed the medication into a dose box), they were dependent on the “hunt.” Challenges related to medication lists becomes even more critical with medications such as warfarin that cannot be dosed by Apodos, (as dosage changes often occur), with the result that the medication list is often adjusted and needs to be updated in the patient’s home. Warfarin treatment requires that a registered nurse measures the patient’s warfarin blood concentration (blood coagulation) regularly and after the test result arrives a new prescription and the dose may need to be adjusted for the patient. This is often a demanding job as the new prescription applies from the same day of testing, and if the patients are not connected to the AC reception (lab at the hospital and where the prescription is visible in the overview accessible to nurses in home health care), that is when the doctor at the health care center prescribes instead of the AC reception, the registered nurses often have to wait for results from there, which often implies a “hunt” for the prescription.

To increase patient safety by reducing the number of delegated drug administrations, the nurses made an effort to get the patients with warfarin (or other medications) on the Apodos system by discussing this with the doctors, and when possible, asking the doctor to change from warfarin to a newer drug that could be dosed by Apodos. They often succeeded, but they also experienced doctors who did not want to put patients on the Apodos system, did not want to replace warfarin for various reasons, and they also had patients who did not want to be on Apodos. This issue jeopardized patient safety in terms of the lack of mutual understanding between health care providers of that each occasion of an additional drug administration meant an extra opening for an adverse advent.

#### Unclear division of responsibilities among health care providers

Deficiencies in the transfer of information are often due to that several health care providers are involved in the patient’s care. Nurses talked about an initiative from the region (county council) called “safe return home” with the aim of improving the transfer of information and follow-ups at home after a patient has been discharged from the hospital, but the nurses saw no major effect of this. The nurses discussed the “safety team” consisting of a registered nurse (together with a physiotherapist, occupational therapist, and assistant nurse) who could be involved up to 4 weeks after the patient’s return from the hospital. The registered nurse then worked in parallel with the home health care registered nurses. Home health care still had the medical responsibility for the patient, while the safety team’s registered nurse acted as a link between the municipality and the hospital. For example, the registered nurse from the safety team did not dispense medicine, so the registered nurses from home health care often had to go home to the patient and dispense medicine immediately after the patient had a visit from the safety team’s registered nurse. However, sometimes the registered nurse dosed the medicine to help the registered nurses in home health care. Engaging the safety team could, consequently, mean even more uncertainties about who is responsible for what, who is responsible for inform the other part, and sometimes the registered nurses in home health care did not get information about that the safety team was involved.

*I do not really understand what she is doing. Sorry but. . . because it has been a bit arbitrary, I have had patients who have been involved with safety teams and then that nurse has dosed the medication but then the next, no then she was not to dose, she was only to change the wound dressing* (nurse 1).

The respondents laughed while comparing the issue of the safety team not dosing medications with that they do not call the social care team if a patient needs to go to the toilet when they are on a home visit. The registered nurses also felt that the goal of the “safe return home” from the hospital could be jeopardized when many different care providers were involved.

### Challenges Related to Delegation

The number of delegations posed many challenges for the nurses, and they were torn between a sense of responsibility for the organization to function, and at the same time responsible for maintaining patient safety. The nurses agreed that they had to delegate the administration of medication to assistant nurses and social care staff as it would not be sustainable if only registered nurses did it. Delegating was crucial to manage the needs of the high number of patients in home health care. *“. . . but the task must be delegated . . . otherwise we will have to work around the clock and go out and give medications to everyone* (nurse 6). For example, nurses delegated the administration of medications, pre-filled injections, insulin, wound dressings, and catheter care.

#### Conveyor-belt delegation

The nurses said that delegating the administration of medications was time-consuming work, especially before the summer when many assistant nurses and social care staff substitutes were employed. Most respondents delegated tasks to many care staff, and they felt that the delegation was sometimes done in a way that was not compatible with patient safety. They felt that they could not verify the knowledge of the delegees to safely administer medication*” Yes, we delegate to three thousand people per year”* (nurse 1)

*Although everyone (nurses) should really only delegate tasks in their own areas, there were so many staff members that it became too much . . . it is not possible to follow up (the delegations), there was no one who could, but one solution was that someone is responsible for all (delegated tasks) . . . but after all, it’s being done in a conveyor-belt manner just to solve the problem* (nurse 4).

There were several issues associated with delegating tasks and patient safety, such as delegating tasks to people who had no experience of health care at all. One of the major problems experienced by the respondents was that many of the social care staff had poor knowledge of the Swedish language. This meant that the nurses could not delegate tasks to those persons, which resulted in staff members not performing one of the major tasks in their area of work.

*Yes . . . well, there are a lot of language barriers now, so sometimes it is a bit difficult to delegate tasks actually . . .. when they do not even write the answers in Swedish . . . what do you write there? “Yes, it was in my language”* (nurse 1).

The managers of the social care groups put pressure on the nurses to delegate tasks as soon as possible to a staff member who was newly employed. Several of the respondents explained that they had delegated tasks to people they were not sure about due to pressure, but that happened mainly when they were new in their nursing role.

*Especially before the summer . . . “he has to be delegated that task now because he will be working nights, so you have to delegate” . . . well and then you try to sit down in a conversation with the person (the recipient of a delegated task) and think about what the person does not have (knowledge/skills), that I will try to give now during this conversation. . .* (nurse 5).

*I have denied several people. . .who have not passed the tests or write very strange answers on the tests. . . and I can understand that because the manager has employed Tom, Dick, and Harry who can hardly speak Swedish, and how do they actually understand the words that are on the test? And I have told the manager that I cannot approve all of them because that is my responsibility* (nurse 6).

The nurses said that a routine was introduced before the summer of 2018. This routine meant that new employees of social care staff would take an online based course via a platform before they took the written delegation test with the registered nurse. The nurses experienced that this resulted in many of the social care staff being more prepared to receive delegation tasks, but staff members remained who did not have the competence for the task or sufficient knowledge of the Swedish language to be delegated medication administration. And given the plan for even more delegations through task shifting from the nurse to the assistant nurse, an extended delegation could mean even more risk to patient safety. This, together with more advanced healthcare, can further jeopardize patient safety.

#### The risks of extended task delegation

The extended delegation of dosing medications to assistant nurses became a discussion in the interviews because some assistant nurses were also delegated to dose medications for 1 week into patient dose boxes. The nurses had different opinions about the appropriateness of assistant nurses dosing medication. The majority felt that the assistant nurses needed knowledge about medications to do that. One respondent said that it was a relief to get help during a time when she was really stressed out. Her colleague then countered with, “*then we go against the constitutions that exist because we are to never delegate due to lack of resources*” (nurse 4*).*

*. . . it’s not just to dose, you get there and then the patient says, “I’ve had such nightmares,” and then I look at the medication list and see that he has Seloken (a beta-blocker), and I know that . . . but the assistant nurse doesn’t know that, and you must have knowledge of medication, so it is not just about dosing.. . . I do not think they should dose, not with the errors that I have seen* (nurse 4).

### Challenges of Advanced Medical Treatments in the Home

Respondents talked about the challenges related to the increased use of advanced medical treatments in patient homes. They suggested that more advanced care at home has become “everyday” because patients are more ill when they are discharged from the hospital. The more advanced medical treatments included intravenous antibiotics for long-term infections, various types of pumps for pain relief, medication for patients with Parkinson’s, medication administration through central venous catheter, total parenteral nutrition, and tube feeding. The nurses felt that more advanced medical treatments in the home requires clarity and structure if it is to be patient safe. They said that a risk assessment must be made before more advanced care measures are implemented in the home. This is because there is less “control” of patients at home than when they are in the hospital.

*A risk assessment must be done. . . and it must be done before they are discharged from the hospital, then there may be challenges that were not known to the discharge unit, and then you must report to the person with medical responsibility that we cannot do that task in a safe way. Then I may not think. . . yes, hygienically speaking, absolutely, but that there is also a risk. . .some patients who may flinch and pull at the tubes, then a risk can arise* (*nurse 2*).

*I’m thinking of this with Central Venous Catheter (CVC) if there is an infection, everything takes a little longer (at home) because you have to call, and the patient has to go to the hospital, and they have to look at it. At home, you change the dressing (CVC) every 7 days, and in the hospital probably every 3 days, there (in the hospital) you see a potential problem faster* (nurse 3).

Nurses also felt that hospital staff did not understand what is required for home care nurses to perform more advanced care in the home environment.

If we get a patient with a Total Parenteral Nutrition (TPN) from the hospital, they do not really understand what it takes for us to be able to do this at home. We need a pump, we need a bag, and we need prescriptions, and they do not really understand that. . .and if it goes wrong we have to consult with a care contact, and it is not always easy to make it work. They just think we solve everything; home care solves everything. But, you have to stand up and say that we cannot take this patient home until everything is ready, and then there are sighs and groans *(nurse 6).*

Collaboration with Hospital-Based Home Care, for which the region is responsible, was another issue the nurses discussed. They felt that the collaboration had improved but was not optimal. One of the respondents called it a “one-way collaboration,” (nurse 3) where the hospital dictated the conditions despite that the threshold principles have established clear rules for when a patient is to be connected to Hospital-Based Home Care.

*I have such a hard time understanding when they, so to speak*, ***choose*** *to take a patient or not because that’s how I experience it. I was recently on a care planning visit with a patient who is palliative, and the doctor (from Hospital-Based Home Care) was only there as a consultant, he said. It was still us (in municipal home health care) who were to conduct all care tasks, and then I think, when do they take the patient and think the patient belongs to them because this patient really did (according to threshold principles) and then I wonder who they choose and how they choose* (nurse 3).

The nurses said that municipal home health care was responsible for basic care and Hospital-Based Home Care for specialized care, but what is included in basic and specialized care was no longer clear as municipal home health care performed more and more advanced tasks in the home. The nurses also stated that Hospital-Based Home Care was very careful to point out that they have the responsibility for the specialized care and home health care for the basic care but what lies within basic and specialized care was experienced to be arbitrarily depending on the patient and the situation and when Hospital-Based Home Care decided to take on the patient or not.

## Discussion

The findings of this study suggest that the main challenges to patient safety related to medication management in municipal home health care in Sweden are challenges related to the transfer of information, challenges in delegating medication tasks and challenges of more advanced medical treatments.

### Information Transfer and Collaboration Between Health Care Providers

The issue of insufficient transfer of information reappeared in the findings and is a well-known threat to patient safety (Lindblad et al., 2018). The nurses struggled with access to information on their patients. Insufficient communication between health care providers has been found to result in fragmented care (Lindblad et al., 2018; Sheehan et al., 2018), that is, the opposite of providing holistic and person-centered care for older people living in the community. One solution that would contribute to more holistic care with increased patient safety is cohesive journaling (Adler et al., 2013). The nurses in this study unanimously agreed that it would be better if all health care providers had the same medical record system. The same medical record system would give all care providers access to the same information about the patients and consequently significantly increase patient safety. A joint medical record system would also reduce cumbersome steps around accessing information. Research has shown that when registered nurses’ routine activities become more complicated and contain obstacles, for example, with patient data entry, the risk of mistakes rises, and patient safety becomes threatened (Yu et al., 2016). As a nurse in home health care, spending time hunting for information also becomes a matter of resources and time efficiency. The registered nurses in home health care seem to spend a lot of time chasing information, not the least information on adjustments to medication lists.

Information between home health care and the hospital did not work well according to the nurses, and the transition of care between hospital and home was highlighted as vulnerable and risky. Previous studies have shown that many adverse events occur soon after discharge from the hospital, and that the majority of these are medical errors that can be avoided (Forster et al., 2004, Madigan 2007; Tsilimingras et al., 2019). Tsilimingras et al. (2019) have found that almost one in every two patients experienced an adverse event after discharge from the hospital. The nurses in this study said that it is a problem when several health care providers are involved in patient care. The initiative “Safe return home” (safety team) was introduced in many Swedish municipalities in conjunction with a law on collaboration in discharge from inpatient care. According to an evaluation of the effects of the law conducted by the SALAR at the end of 2018, collaboration between care providers had improved, but there was limited knowledge about improvement in security (SALAR, 2018). Another problem identified by the nurses in this study was the collaboration with Hospital-Based Home Care on the care of patients who need specialized care. They stated that the region has interpretive precedence, and the municipality must comply with the hospital’s decision to take on the patient or not. This could reflect a power relationship between the hospital and the municipality which must be erased since it risks affecting patient’s right to equal care and patient safety. This may also reflect an outdated view of municipal health care that no longer corresponds to reality. In addition, there is a history of hospitals having high status, and it has also been considered higher status to work as a nurse at a hospital than to work for the municipality. This pre-understanding of community health care exists even though a nurse employed by a municipality is required to have extensive knowledge and be prepared for any acute care situation. The background to the perception of municipal employment as low status can be difficult to sort out. The status can be related to the patient group or the place where the care is provided. The status can also result from a combination of the two. However, the problem seems to permeate the health care system, even patient outcomes. It would therefore be interesting to study how these power relations affect patient safety and patient care.

The collaboration between regions and municipalities is a hot topic in Sweden. Care is to be based on a patient’s diagnosis and needs and not on medical equipment or basic and/or specialized care. The most important thing is the patient, not if the person providing care is employed by a municipality or a region (Swedish Government, 2020). Given the transition to “Nära vård” and the decentralization of care from hospitals, home health care will become even more advanced than it is today. With more advanced health care tasks come new and more situations where errors and injury can occur for patients (Yu et al., 2016). The respondents said that it is impossible to have the same type of control in a home as in a hospital because medical staff are not constantly present in the home. We therefore suggest research on how to ensure that health care provided “close” is as patient safe as that provided in hospitals.

### Delegations and Regulations

On January 1, 2018, HSLF-FS 2017: 37 (Swedish Government, 2017) entered into force in Sweden. The main changes in the regulation were that the same rules applied regardless of where the care takes place. This means that patient safety standards must be the same regardless of whether the medication is handled at an institution or at home. A huge difference between hospital care and home health care is that registered nurses do not delegate the administration of medications to unlicensed care staff in hospitals. According to the World Health Organization (2017), most medical errors occur via the administration of medications, an area that is delegated to assistant nurses and social care staff by nurses in home health care. The prerequisites for patient safety are thus not comparable.

The delegation of tasks in home health care is part of an ongoing task-shifting process, which is currently taking place within home health care (Odzakovic et al., 2021). The dosing of medication (weekly, in dose boxes), not only the administration, can also be delegated to social care staff. Extended delegations were considered as threats to patient safety and the respondents discussed the underlying reasons such as to relieve the nurse’s duties and time consuming tasks. They stated that nursing skills were needed to link the symptoms and patient well-being with the medication therapy and delegations should always be based on the patient’s needs and must never be done at the expense of patient safety. At the same time, our findings indicate that delegating tasks in home health care is inevitable, which is in accordance with the findings of (Craftman et al., 2013) who states that the law of delegating on patients’ needs is placed in a subordinate position. The nurses felt that home health care would not work without delegating the administration of medications to unlicensed care staff, and that there sometimes was uncertainty about whether the person had the competence to perform a delegated task. As they had to “accept” that these delegations had to be made in order to handle the large number of patients, they made efforts to reduce the number of tasks where medical errors can occur, and where patient safety is threatened.

The nurses experienced pressure to delegate medication administration, especially when there were summer substitutes among social care staff. The pressure came from social care managers who need their groups to be staffed with people who have delegation. The impact of leadership on patient safety has been discussed. Leaders have a key role in designing, encouraging, developing, and communicating patient safety culture (Ree & Wiig, 2020). The problems with pressure from the social care manager could be attributed to a lack of knowledge about the registered nurse’s work in home health care. Most social care managers are qualified in social welfare and may have difficulty understanding the nursing profession (Nilsson et al., 2009). However, it is the care provider’s responsibility and not the nurses to ensure that staff members have the right competence. According to our findings, assessment of competence, still seems to be the responsibility of the registered nurse when the care provider (social care staff manager) sometimes employs people with non-existent care experience and sometimes even poor Swedish language skills. Consequently, registered nurses must ensure that patients receive medications, and at the same time they must ensure that the person who administers the medications has the right competence.

The findings of this study resulted in an overall reflection at an organizational and fundamental level of patient safety related to medication management in home health care. Is it possible to improve collaboration between care providers when there is insufficient knowledge about the involved organizations between the care providers participating in a collaboration? Knowledge of your partners’ conditions and resources should be a cornerstone of effective collaboration. There seems to be a huge gap in understanding and insight into home health care as an organization. These issues are important as the lack of knowledge about a registered nurse’s work, conditions, and resources not only makes it difficult for the nurse to carry out their work, it also affects medication management and overall patient safety. Working as a registered nurse in municipal home health care has previously been described as working in a black box (Odzakovic et al., 2021). Lack of transparency in an organization may also create patient safety problems. When nurses in home health care work in isolation, when there is no control system, that is, no colleagues present (Nilsson et al., 2009), the patient’s vulnerability increases along with the risk that the patient is exposed to malpractice.

### Strengths and Limitations

This study contributes with important knowledge about patient safety issues related to medication management in home health care. However, there are limitations to the study that should be addressed. The main criticism of the use of vignettes has been the possible difference between the vignette and social reality (Barter & Renold, 1999). We used vignettes from concrete real-life situations in the context where the respondents’ work to increase the credibility of the results and the vignettes reflected an everyday (working) life that the respondents recognized. Hence, when the vignette represented a typical situation, it provided an intersubjectivity on a group level, as a gradual increase of the intersubjective process. The recognition of the phenomenon among nurses can thus be seen as an intermediate step, as a part of the process toward an objective and expanded description of subjective meaning (Schuetz, 1953). However, the vignettes proved to be credible and recognizable to the respondents, which entails the trustworthiness of the results.

The use of vignettes can be considered as aiming the phenomenon in a certain direction. One could therefore discuss whether it can be considered as an inductive analysis when vignettes are based on situations and events in which the relevance of the vignette could either be confirmed or denied. The use of vignettes as predetermined themes for discussion may limit discussions of other aspects related to medication management. To avoid limiting perspectives to the topics of the vignettes, we strived to gain new/other perspectives through follow-up questions and by staying as neutral to the vignette as possible. If the nurses could not relate to the vignette, it was emphasized that it was equally important to gain information of why they could not relate. The nurses were also asked if they wanted to add anything else related to the vignettes or outside the context of the vignettes.

Recruitment of nurses was challenging. This may explain why there are few studies on the subject. Initially we planned to conduct group interviews with nurses from different home care units. The data would have been richer if the registered nurses from the different units had been able to discuss similarities and differences between the units. Only six nurses participated in the study, and one of the units was represented by the perspectives of one nurse while the other two units were based on two or three nurses’ perspectives, which is another limitation to the study.

In addition, our study took place in a Swedish context of health care which can be considered to limit transferability. However, bearing in mind the increasing numbers of older people with complex care needs worldwide and a rise of home health care in many countries, we believe that our findings are applicable to the current development and adaptions of health care to an international level.

## Conclusions

The issue of transfer of information permeated all parts of our findings. Coordinating patient medications, delegating tasks, and more advanced care require clear communication between the parties concerned to be compatible with patient-safe care. It is undisputable that a joint medical record system for all health care providers would make it easier for nurses in home health care to be able to control and ensure that patients receive correct medication therapy and that all care providers have the same information about the patients and their medications. Delegations compatible with patient safety are another area that is difficult to control for the nurse in home health care and that needs to be improved with regard to ensuring that the person receiving the delegation has the right competence to perform the task. An additional important lesson and clinical implication from this study is that knowledge about and insight into a registered nurse’s work in home health care is crucial for improved collaboration between care providers. Increased knowledge of home health care on the part of other care providers could lead to them taking greater responsibility to communicate the information that they know is missing and necessary for the registered nurses in home health care to perform their work with greater patient safety.
